# Exploring the Diagnostic Odyssey of IgA Vasculitis

**DOI:** 10.7759/cureus.68170

**Published:** 2024-08-30

**Authors:** Irene Rosmaninho, Beatriz Simão-Parreira, Carolina Leal, Leila Cardoso, Jorge Almeida

**Affiliations:** 1 Internal Medicine Department, Unidade Local de Saúde de São João, Porto, PRT; 2 Anatomopathology Department, Unidade Local de Saúde de São João, Porto, PRT

**Keywords:** cutaneous biopsy, kidney biopsy, leukocytoclastic vasculitis, palpable purpura, iga vasculitis

## Abstract

IgA vasculitis is a form of small vessel vasculitis characterized by IgA immune complex deposition. Although primarily affecting children, it can also occur in adults, often with more severe manifestations and a higher risk of chronic kidney disease (CKD). The authors present the case of a 77-year-old man with significant cardiovascular risk and atrial fibrillation who was admitted to the emergency department (ED) with pruritic and painful palpable purpuric rash, weight loss, and asthenia persisting for approximately one month prior to presentation. Laboratory findings revealed normocytic normochromic anemia, worsening renal function, elevated inflammatory markers, and leukoerythrocyturia. Initially diagnosed as infectious purpura associated with urinary tract infection, the patient was discharged with a prescription for antibiotics (cefixime). Subsequent worsening of the skin lesions, constitutional symptoms, and gross hematuria prompted a second visit to the ED, and the persistent deterioration of kidney function and inflammatory parameters led to admission for further investigation and consideration of a kidney biopsy. This case report describes the etiological investigation, providing a brief review of the typical characteristics of this disease and highlighting the importance of certain factors in establishing the diagnosis, notably the need and timing of a biopsy of the affected organ for a definitive diagnosis. Additionally, the clinical case underscores the diagnostic challenge, particularly when histological confirmation is elusive.

## Introduction

IgA vasculitis, formerly known as Henoch-Schönlein purpura, is a small vessel vasculitis mediated by the deposition of IgA-dominant immune complexes. It is the most frequent vasculitis in children, with its incidence declining in adulthood, where it usually presents as a more severe disease and a higher risk of progression to chronic kidney disease (CKD) [1].

Most cases of IgA vasculitis are sporadic, and its etiology remains largely unknown. Recent investigative studies suggest that around 10% of cases may be associated with genetic defects [namely in C1GALT1 (a galactosyl transferase enzyme) and C1GALT1C1 (a molecular chaperone)] [2]. Also, a common pathogenic pathway with IgA nephropathy is accepted, with infections being the most frequent triggers, justifying its seasonality. Clinically, it presents with a classic tetrad of symptoms: palpable non-thrombocytopenic purpura, arthralgias or arthritis, abdominal pain, and renal dysfunction, with the most consensual diagnostic criteria being the one proposed by the European Alliance of Association for Rheumatology (EULAR) and the Paediatric Rheumatology European Society (PRES), where the presence of petechiae or purpura in the absence of coagulopathy or thrombocytopenia is mandatory. Renal involvement is a significant complication, with the deposition of IgA immune complexes in the glomeruli leading to inflammation, and it is the main prognostic determinant. Histologically, IgA vasculitis is characterized by leukocytoclastic vasculitis and IgA deposition in affected tissues [3]. In this report, we present the case of a patient admitted with palpable purpura, detailing the diagnostic and etiological investigation and offering a brief review of the typical disease characteristics.

## Case presentation

We present a 77-year-old white male with hypertension, dyslipidemia, obesity, and hyperuricemia. Additionally, he had atrial fibrillation and ischemic heart disease, having previously undergone percutaneous coronary intervention. His medical records also include normochromic normocytic anemia of unknown etiology, KDIGO IIIb-grade chronic kidney disease, and benign prostatic hyperplasia. Current medications consisted of edoxaban 60 mg, bisoprolol 10 mg, lisinopril 10 mg, and atorvastatin 20 mg.

The patient presented to the emergency department (ED) and was referred by a primary care physician (PCP) due to the appearance of purple lesions on his lower limbs and asthenia. He reported the onset of lesions in the prior month, initially manifesting on his thighs and progressively worsening over the five days through his legs and feet. These purpuric lesions were painful and itchy and impeded his ability to walk. Additionally, he experienced marked asthenia and unintentional weight loss of approximately 20 kg over the last year and a half. He mentioned a recent episode of self-limited epistaxis and a small amount of rectal bleeding following a painful bowel movement about 15 days before admission. Physical examination confirmed extensive purpuric lesions, mainly on his legs, which were pruritic, tender to touch, non-blanching, and associated with mild malleolar edema. Analytical findings showed a slight exacerbation of previously documented anemia (hemoglobin 10.8 g/dL), worsened renal function (serum creatinine 1.88 mg/dL, glomerular filtration rate (GFR) 34 mL/min/1.73 m^2^; basal serum creatinine 1.3 mg/dL, GFR 49 mL/min/1.73 m^2^), elevated inflammatory markers, and active urinary sediment with leukocytes and erythrocytes, without nitrites (Table 1). At this juncture, the patient was diagnosed with non-thrombocytopenic purpura associated with urinary tract infection and was discharged with a prescription for cefixime 400 mg. The attending physician recommended a follow-up assessment after three to four days to potentially adjust the treatment based on the urine culture results and reassess renal function after the resolution of the infection.

**Table 1:** Comparison of analytical and imaging studies between first and second admissions to the emergency department Hb: hemoglobin; MCV: mean corpuscular volume; MCHC: mean corpuscular hemoglobin concentration; WBC: white blood cell count; Neut: neutrophils; Creat: serum creatinine; CRP: C-reactive protein.

Parameter	Reference value	First admission	Second admission	
Hb (g/dL)	13.0–18.0	10.8 (11.3)	9.0	
MCV (fL)	87.0–103.0	94.3	94.3	
MCHC (g/dL)	28.0–36.0	32.4	31.8	
WBC (× 10^9^/L)	4.0–11.0	8.62	9.08	
Neut (%)	53.8–69.8	76.6	80.1	
Urea (mg/dL)	10.0–50.0	72.0	79.0	
Creat (mg/dL)	0.67–1.17	1.88 (1.3)	1.82	
CRP (mg/L)	<3.0	117.7	118.1	
Urine analysis				
Nitrites		Negative	Negative	
Leucocytes		703.6/μL	25237.5 /μL	
Erythrocytes		161.1 /μL	25205.4 /μL	

**Figure 1:**
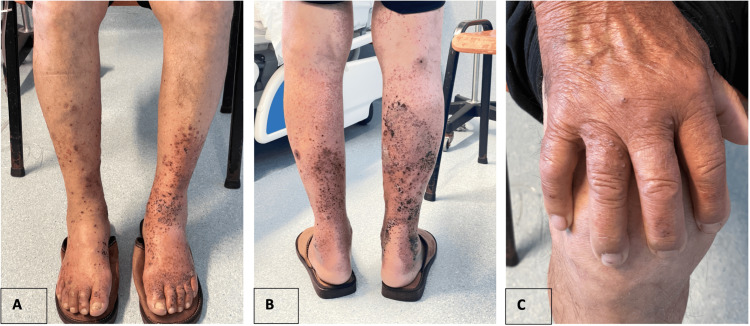
(A-C) Skin lesions on admission to hospitalization Legend: Small, raised, reddish-purple spots on legs (A-B) and hands (C) of the patient on admission, constituting palpable purpura.

Twelve days after discharge, the patient was readmitted to the ED due to gross hematuria. He reported worsening lower limb lesions and complained of terminal hematuria lasting approximately five days, decreased urinary output, and dizziness for the past two days. He denied new pain complaints, urinary symptoms, or fever. During this admission, there were no episodes of hypotension, and the patient's urinary output was maintained. Analytical studies revealed worsened anemia (hemoglobin 9.0 g/dL), persistent renal dysfunction, and elevated inflammatory parameters. Repeat urine analysis showed persistent leukocyte and erythrocyte presence. A renal ultrasound was performed, which demonstrated normal-sized kidneys with lobulated contours and areas of focal reduction in cortical thickness, likely scarring in nature. The corticomedullary differentiation was relatively preserved bilaterally, and no hydronephrosis was detected bilaterally. Thus, renal obstruction was ruled out, and the patient demonstrated improved renal function following fluid resuscitation (serum creatinine from 1.82 to 1.33 mg/dL). Given the complexity of the presentation, a multidisciplinary approach involving nephrology and dermatology was undertaken, with both specialties recommending further inpatient evaluation and possible biopsy. Subsequently, the patient was admitted to the Internal Medicine Service for further evaluation and management. The skin lesions on admission to hospitalization are illustrated in Figure 1(A)-1(C).

On the second day after admission, we conducted a comprehensive assessment of the patient's condition, noting complaints of weight loss of approximately 20 kg over the past year and a half, along with asthenia. The patient denied any visual or auditory issues and was experiencing respiratory, cardiovascular, urinary, gastrointestinal, or neurological symptoms. However, he did report long-standing bilateral knee pain characterized by mechanical symptoms associated with known degenerative osteoarticular pathology. From the study conducted, attention is drawn to the worsening of the previously known anemia (Table 1), along with iron deficiency and the absence of vitamin B12 or folate deficiency, thrombocytopenia, or leukocytosis; elevation in inflammatory parameters, namely sedimentation rate and C-reactive protein; normal coagulation tests; and resolution of renal dysfunction after instituted fluid therapy. As a result, the hypothesis of palpable purpura associated with thrombocytopenia or coagulation disorders was excluded, and we considered the possibility that the palpable purpura could have an infectious or post-infectious origin, given the recent treatment for a urinary infection. However, no microbiological isolation was obtained from urine or blood cultures collected previously in the emergency department or during hospitalization. Additionally, other chronic infections were ruled out as potential causes (Table 2).

During the hospitalization, a multidisciplinary team consisting of internal medicine, dermatologists, and nephrologists managed the patient. Dermatology assessed the skin lesions, suggesting a diagnosis of leukocytoclastic vasculitis in a probable infectious context. However, confirming the diagnosis histologically was deemed unnecessary, and topical corticosteroid therapy was initiated due to the absence of involvement of other skin organs. Nephrology considered vasculitis as a possible diagnosis, but the rapid improvement in renal function post-fluid therapy made renal damage less likely. Nevertheless, further investigation was warranted to explore other organ involvement and determine the etiology. To assess renal involvement, a repeat urine test was requested, which showed leukocytes and dysmorphic erythrocytes in the sediment, and the 24-hour urine analysis revealed subnephrotic proteinuria (1.85 mg/24 h). Evaluation of respiratory tract involvement, despite the absence of symptoms, included chest and sinus computed tomography (CT) scans, which showed no significant abnormalities.

Given the persistent constitutional symptoms, paraneoplastic, infectious, and inflammatory/autoimmune etiologies were considered and investigated accordingly. A head CT, thoraco-abdomino-pelvic CT, and a full-body PET scan (positron emission tomography) were requested and performed sequentially. These imaging studies revealed no lesions suggestive of malignant neoplasia. 

In view of the presence of macroscopic hematuria, an evaluation by a urologist was requested, and a cystoscopy was performed, which ruled out the presence of endovesical neoplasms. Due to worsening anemia and the previous history of gastrointestinal bleeding, upper and lower digestive endoscopies were conducted. However, no visible endoscopic lesions were observed.

At this point, an extensive workup was carried out (Table 2), revealing slightly elevated IgA levels (564.0 mg/dL, normal range: 90.0-410.0 mg/dL) and positive anti-nuclear antibodies (ANA) at titers of 1/320 with a mottled pattern and 1/640 with an intercellular bridge pattern. There was no consumption of complement factors, and ANCA PR3, ANCA MPO, anti-cardiolipin antibodies, rheumatoid factor, cryoglobulins, and circulating immune complexes were all negative.

**Table 2:** Background information of study cohort consisting of orthodontic patients. Hb: hemoglobin, MCV: mean corpuscular volume, MCHC: mean corpuscular hemoglobin concentration, WBC: white blood cell count, Neut: neutrophils, Ret: reticulocyte count, Hpt: haptoglobin, FA: folic acid, VitB12: vitamin B12, Fe: iron, TSAT: transferrin saturation, Ferr: ferritin, TP: total proteins, Alb: albumin, Urea: blood urea nitrogen, Creat: serum creatinine, CRP: C-reactive protein, ESR: erythrocyte sedimentation rate, VitD: vitamin D, IgA: immunoglobulin A, PSA: prostate-specific antigen, C3c: complement component 3c, C4: complement component 4, CH50: complement hemolytic activity, ANA: antinuclear antibodies, Anti-Ds DNA: anti-double-stranded DNA antibody, ANCA: antineutrophil cytoplasmic antibodies, Anti-GBM: anti-glomerular basement membrane antibody, Anti-CL: anti-cardiolipin antibodies, RF: rheumatoid factor. (1) Autoimmune markers include anti-GBM antibody, anti-CL Antibodies, anti Jo-1, anti RNP, anti Scl-70, anti Sm, anti Ssa, anti SSb, lupus inhibitor, cryoglobulin, and circulating immune complexes. (2) Bacterial and viral serology include borrelia antibody (Burgdoferii, Garinii, Afzelii), Rickettsia conorii antibody, Wright reaction, Coxiella burnetti antibody, and EBV antibodies (IgM CVA, IgG Early).

Variable	
Median age (25^th^ and 75^th^ percentiles)	21 years (15–29 years)
Sex (proportion)	
Female	35 (76.1%)
Male	11 (23.9%)
Where in treatment	
Pre-treatment	5 (10.9%)
In active treatment	28 (60.9%)
In retention	11 (23.9%)
Other	2 (4.4%)
Types of treatment	
Before treatment	1 (2.2%)
Fixed appliance	36 (78.3%)
Aligner	3 (6.5%)
Functional appliance	2 (4.3%)
Other	4 (8.7%)

In summary, we have a patient presenting with complaints of palpable purpura, gross hematuria, asthenia, and weight loss. Upon extensive evaluation, normochromic normocytic anemia of unknown etiology, elevated inflammatory markers, and AKIN (acute kidney injury network) 1 acute kidney injury promptly corrected with fluids were identified, along with active urinary sediment and subnephrotic proteinuria. Further investigations, including imaging studies and endoscopies, were conducted, ruling out neoplastic lesions. The comprehensive blood workup revealed elevated IgA levels and positive anti-nuclear antibodies and excluded other autoimmune and infectious causes. Therefore, after a second multidisciplinary discussion, it was deemed crucial to perform a skin and kidney biopsy in an attempt to reach a definitive diagnosis.

**Figure 2:**
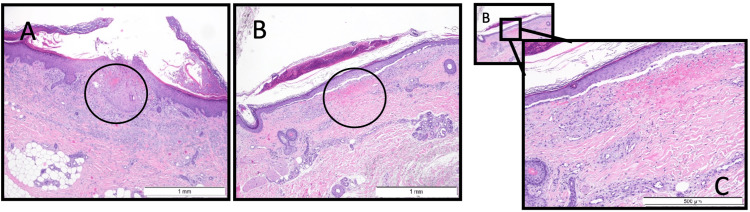
(A-C) Skin biopsy Epidermal blister with cellular debris. In the adjacent dermis, there is erythrocyte extravasation and focal necrosis (A-B), as well as capillary proliferation (C). Fibrinoid necrosis of blood vessel walls and perivascular neutrophilic infiltrate (C) consistent with leukocytoclastic vasculitis, findings suggestive of vasculitis.

**Figure 3:**
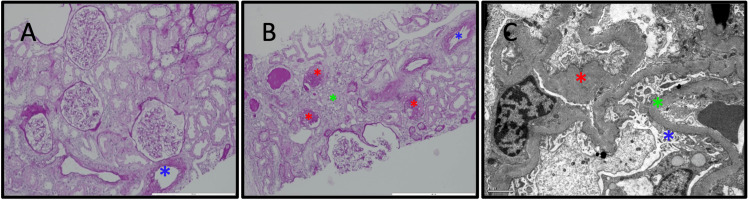
(A-C) Renal biopsy Acute tubular necrosis lesions and interstitial fibrosis (A-B): arteriosclerosis marked with a blue asterisk; fibrosis and atrophy marked with green; and sclerosed glomerulus with red. Immunofluorescence findings (C): glomerular sclerosis marked in red; glomerular basement membrane in blue; fusion of pedicels marked in green. No immune deposits were found at electron microscopy.

**Figure 4:**
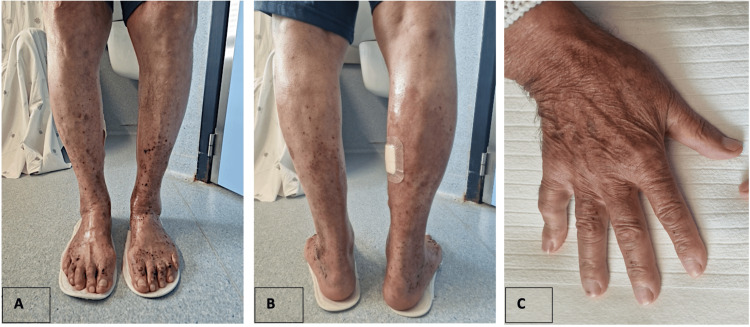
(A-C) Purpuric lesions at discharge Purpuric lesions of legs (A-B) and hands (C) at discharge.

The skin biopsy (Figure 2) revealed intracorneal vesicle-blister and pustule/horny crust lesions with erythrocyte extravasation and signs of old hemorrhage with no vasculitic lesions present. In contrast, the renal biopsy (Figure 3) showed podocyte pedicel fusion and glomerular basement membrane thickening, with immunofluorescence revealing light staining for IgA, C3c, and Lambda at the mesangial level. Despite these frustrating findings, most likely related to the delay in performing the biopsy, IgA vasculitis emerged as the most probable diagnosis considering the clinical presentation and investigative findings. The prognosis for IgA vasculitis is generally good, with most cases resolving within several weeks to months. However, complications such as nephritis can occur, leading to chronic kidney disease in some cases. Given that the patient has improved skin lesions (Figure 4) and resolved kidney dysfunction, he was discharged on the 19th day of hospitalization, and we kept monitoring him as an outpatient for any recurrence of symptoms, particularly renal involvement, which may require ongoing nephrological care.

As a complication during hospitalization, the patient complained of bilateral shoulder pain and stiffness with an inflammatory pattern suggestive of polymyalgia rheumatica, consistent with the PET scan results. After properly excluding giant cell arthritis, treatment with prednisolone 20 mg daily was initiated, leading to rapid resolution of symptoms. Throughout his hospitalization, the patient remained hemodynamically stable and afebrile, with no symptoms of dizziness, respiratory, urinary, or gastrointestinal complaints. Inflammatory parameters remained persistently high despite the resolution of symptoms and corticosteroid therapy, and hematic traces were intermittently detected in the urine. The skin lesions progressively improved under the application of topical corticosteroid therapy (Figure 4).

Furthermore, managing the patient's underlying comorbidities, such as hypertension, dyslipidemia, and chronic kidney disease, was essential for overall health maintenance. After the resolution of acute kidney injury (at discharge, serum creatinine 1.19 mg/dL, GFR 58) and considering the most likely diagnosis of IgA vasculitis, he resumed his usual treatment with anticoagulation and lisinopril. Oral supplementation with cyanocobalamin, folic acid, and cholecalciferol was initiated to address the targeted vitamin deficiencies. The patient continued daily application of emollients and topical corticosteroid therapy with betamethasone ointment, as recommended by dermatology. Since discharge, close follow-ups as an outpatient at the Internal Medicine and Nephrology consultation with regular check-ups and monitoring of renal function and blood pressure have been implemented. The patient remains stable, with no recurrence of skin lesions or kidney dysfunction (serum creatinine of 1.3 mg/dL, GFR 49 mL/min/1.73 m^2^).

## Discussion

When initially approaching a patient with palpable purpura, it is crucial to consider systemic vasculitis among the differential diagnoses. Palpable purpura can be a manifestation of various vasculitic syndromes, including IgA vasculitis (formerly known as Henoch-Schönlein purpura), granulomatosis with polyangiitis, microscopic polyangiitis, and eosinophilic granulomatosis with polyangiitis [4,5]. These conditions often present with systemic symptoms such as fever, arthralgias, and constitutional symptoms alongside skin lesions. Differential diagnosis also includes coagulopathies, infectious diseases such as meningococcemia, drug reactions, and other causes of cutaneous vasculitis. A comprehensive clinical evaluation, including a detailed medical history, physical examination, and appropriate laboratory tests, must differentiate between these etiologies and guide further management. A skin biopsy may be required to confirm the diagnosis and determine the specific type of vasculitis based on histological findings.

IgA vasculitis is characterized by small vessel vasculitis mediated by the deposition of IgA-dominant immune complexes. Although more common in pediatric populations, it can occur in adults, with an annual incidence of 0.1 to 1.8 per 100,000 individuals [6,7], as observed in our case. The disease shows a slight predilection for males, with a male-to-female ratio of approximately 1.5 [8]. Additionally, IgA vasculitis tends to manifest more frequently in certain age groups, with peaks of incidence observed in children aged four to six years and adults aged 50 to 70. These demographic trends highlight the importance of considering IgA vasculitis as a potential diagnosis across different age groups, particularly in adults presenting with characteristic suspected clinical features.

The exact pathological mechanism of IgA vasculitis remains incompletely understood, but it is believed to involve aberrant immune responses triggered by various antigenic stimuli. These stimuli can include infectious agents such as group A streptococcus, Helicobacter pylori, parvovirus B19, hepatitis B virus, human immunodeficiency virus (HIV), and Stenotrophomonas maltophilia, among others. Additionally, allergens, tumor antigens associated with malignancy, and autoimmune diseases have been proposed as potential triggers for IgA vasculitis. The pathophysiology involves increased IgA synthesis, which may be related to exposure to antigens processed by the mucosa-associated immune system. This dysregulated immune response leads to the formation of IgA immune complexes that deposit in small blood vessels, triggering inflammation and tissue damage. The deposition of IgA immune complexes primarily affects the skin, joints, gastrointestinal tract, and kidneys, resulting in the characteristic clinical manifestations of palpable purpura, arthralgias or arthritis, abdominal pain, and renal dysfunction. Further research is needed to elucidate the precise mechanisms underlying IgA vasculitis pathology and to develop targeted therapeutic strategies [3,9,10].

The clinical presentation of IgA vasculitis typically involves a tetrad of symptoms: palpable purpura, arthralgia/arthritis, abdominal pain, and renal dysfunction. Skin involvement is most common; lesions typically present as palpable purpura, which are small, raised, reddish-purple spots on the skin. These spots can vary in size and are caused by bleeding underneath the skin due to inflammation of the blood vessels. Additionally, patients may develop ecchymosis, which is larger areas of bruising, and petechiae, which are tiny red or purple spots caused by small amounts of bleeding. These skin manifestations are often accompanied by other symptoms such as joint pain, gastrointestinal issues, and kidney involvement. While the skin lesions are a hallmark feature of IgA vasculitis, they usually resolve within a few weeks without scarring. However, in some cases, they may recur or persist longer. Prompt recognition and management of IgA vasculitis are crucial to prevent complications and ensure optimal patient outcomes.

Renal and gastrointestinal manifestations are two other common and significant disease features. Renal involvement typically presents with symptoms such as hematuria, proteinuria, and sometimes nephrotic syndrome. Renal biopsy findings often reveal IgA deposition in the glomeruli, indicative of IgA vasculitis nephritis. Approximately 30-50% of patients with IgA vasculitis experience renal involvement, which can range from mild, self-limiting disease to severe, potentially leading to chronic kidney disease [3,5,8,11]. Gastrointestinal involvement manifests with symptoms like abdominal pain, nausea, vomiting, and occasionally gastrointestinal bleeding. The most serious complication is intussusception, requiring urgent medical intervention. 

Furthermore, IgA vasculitis can involve the respiratory system, manifesting as cough, hemoptysis, or pulmonary infiltrates on imaging studies. Less frequently, the central nervous system may be involved, resulting in headaches, seizures, or focal neurological deficits. While these manifestations are less common compared to renal and gastrointestinal involvement, they underscore the systemic nature of IgA vasculitis, necessitating comprehensive evaluation and management to address potential multi-organ complications.

Diagnosing IgA vasculitis is challenging due to the absence of specific diagnostic tests. Instead, clinicians rely on clinical criteria and laboratory studies to assess disease severity and exclude other potential diagnoses. Endoscopic studies may be utilized to identify gastrointestinal involvement, while renal biopsy can confirm glomerular pathology. However, it is important to note that histological findings may not always align with clinical suspicion, emphasizing the need for careful clinical correlation in the diagnostic process.

The EULAR proposed diagnostic criteria for IgA vasculitis, which are primarily used in pediatric populations but serve as a guide for adult diagnosis as well. These criteria, revised in 2006, have demonstrated high sensitivity and specificity in diagnosing Henoch-Schönlein purpura (HSP), with a sensitivity of 99.2% and specificity of 86% [12,13]. The criteria include the mandatory presence of purpura or petechiae predominantly in the lower limbs without thrombocytopenia or coagulopathy, along with at least one of the following: abdominal pain, arthritis or arthralgia, renal involvement with proteinuria or hematuria, and histological evidence of leukocytoclastic vasculitis or proliferative glomerulonephritis with predominant IgA deposition. These guidelines provide a structured approach to diagnosing IgA vasculitis and help ensure early recognition and appropriate management to improve patient outcomes.

Therefore, diagnosing IgA vasculitis requires a multifaceted approach that incorporates clinical assessment, laboratory studies, imaging, and sometimes tissue biopsy. The most frequent analytical findings in IgA vasculitis typically include elevated serum IgA levels, inflammatory markers, and evidence of renal involvement, such as hematuria and proteinuria. Imaging studies, such as ultrasound or CT scans, may be performed to assess the extent of organ involvement, particularly in cases where abdominal symptoms are prominent. In some instances, a skin or renal biopsy may be necessary to confirm the diagnosis, although the typical histological results may be absent. Various infectious agents have been implicated as potential triggers for the disease and should be investigated when clinically pertinent. Research suggests a potential association between IgA vasculitis and malignancy, particularly solid tumors or their metastases. While lympho and myeloproliferative neoplasms are more common in vasculitis cases in general, IgA vasculitis demonstrates a greater association with solid tumors. This association may manifest before, during, or after the diagnosis of cancer, highlighting the need for additional investigations to screen for occult neoplasia in adult patients with IgA vasculitis [3,5,9,14]. Additionally, there is evidence suggesting a role for genetic factors in predisposing individuals to developing IgA vasculitis. Certain genetic polymorphisms associated with the immune system, particularly those involving the IgA pathway, have been implicated in increasing susceptibility to the disease. However, the precise relevance of this contribution remains poorly understood and warrants further investigation [15].

Despite advances in understanding IgA vasculitis, several diagnostic and therapeutic uncertainties persist. Even with extensive evaluation, cases like the one described may remain diagnostically elusive, leading to frustration for both clinicians and patients. In such instances, performing kidney and skin biopsies becomes imperative to shed light on the underlying pathology. The timing and quality of biopsies can significantly impact diagnostic accuracy, especially if performed during the resolution phase of skin lesions or in the absence of active renal pathology.

Skin biopsy, particularly from the edge of a recent lesion, can reveal histological features characteristic of IgA vasculitis. The most typical histologic finding is leukocytoclastic vasculitis, characterized by an inflammatory infiltrate predominantly consisting of neutrophils and monocytes. Additionally, foci of fibrinoid necrosis in the vessel wall, occasional thrombosis, and erythrocyte extravasation may be observed, accompanied by predominant IgA deposition [16]. Although skin biopsies may not always confirm the diagnosis definitively, they provide valuable insights into the pathophysiology of the disease and help guide further management. Similarly, renal biopsy is crucial in diagnosing IgA vasculitis, especially in cases with renal involvement. Histological examination of renal tissue can reveal changes ranging from isolated mesangial proliferation with mesangial IgA deposits to severe crescent glomerulonephritis. These findings not only aid in confirming the diagnosis but also provide valuable prognostic information regarding the severity of renal involvement and the risk of long-term complications. Thus, while the diagnostic journey for IgA vasculitis may be fraught with challenges, kidney and skin biopsies serve as invaluable tools in elucidating the underlying pathology and guiding appropriate management strategies.

Given the patient's history of a recently treated urinary infection, an initial hypothesis of infectious/post-infectious vasculitis was considered. However, the lack of microbiological evidence and the persistence of symptoms led to further investigation. Therefore, a thorough clinical history and organ-system review, coupled with diagnostic tests, were crucial not only for establishing a diagnosis but also for detecting potential life-threatening complications early on.

This patient’s clinical features, including constitutional symptoms like asthenia and weight loss, worsening normocytic normochromic anemia, and complaints of hematuria and rectal bleeding, prompted immediate consideration of paraneoplastic vasculitis. However, thorough analytical and imaging studies ruled out neoplastic processes, emphasizing the need for a meticulous diagnostic approach. On the other hand, the presence of palpable purpura lesions concomitant with renal involvement, evidenced by hematuria and proteinuria, along with elevated serum IgA levels, strongly supported the diagnosis of IgA vasculitis. However, due to the rapid improvement of renal function and the incomplete clinical picture, after careful deliberation and considering the necessity to achieve a definite diagnosis, we decided in favor of obtaining skin and kidney biopsies.

Skin biopsy findings revealed purpuric lesions devoid of vasculitis lesions or immune deposits upon immunofluorescence study. In contrast, renal biopsy indicated features consistent with acute tubular necrosis alongside nonspecific changes suggestive of chronicity. As previously discussed, timing and biopsy quality are critical factors influencing diagnostic accuracy, especially when performed during the resolution phase of skin lesions, after starting treatment, or in the absence of active renal pathology. In our case, the skin biopsy was performed 13 days after treatment with topical corticosteroids, and the renal biopsy was performed 17 days after admission. One possibility for the current results is that they could indicate a mild or rapidly resolving IgA vasculitis, which might contribute to inconclusive biopsy results, especially when considering delayed sampling.

The dynamic nature of IgA vasculitis accentuates the reliance on clinical judgment in interpreting diagnostic tests and guiding therapeutic decisions. Another diagnostic dilemma in IgA vasculitis is differentiating it from other systemic vasculitides, particularly in adults where disease criteria EULAR serve mainly as a guide but may not capture the full spectrum of disease variability. Despite inconclusive biopsy results, clinical suspicion remains pivotal, particularly in cases lacking histological confirmation. In our patient, the concurrent involvement of multiple organs, including the skin and kidneys, provided additional support for the diagnosis.

Once diagnosed, the management of IgA vasculitis hinges on balancing symptom control with minimizing long-term complications. Supportive measures such as hydration, rest, and pain management are essential, but the role of immunosuppressive therapy remains controversial [5]. While systemic glucocorticoids may be warranted in severe cases, their use is not without risks, especially in the context of potential adverse effects and variable treatment response [8,11,17,18].

Renal involvement poses a significant prognostic factor in IgA vasculitis, highlighting the importance of early detection and intervention. ACE inhibitors and ARBs have shown efficacy in preserving renal function and reducing proteinuria, mainly in patients with proteinuria >0.5 g/day, but their optimal timing and duration of use remain uncertain. Similarly, the role of immunosuppressive agents in preventing disease progression warrants further investigation, especially in patients with severe renal involvement. Patients with proteinuria >1 g/day should be started on corticosteroids, and those with more than 20-25% active crescents on renal biopsy should be treated with cyclophosphamide, mycophenolate mofetil, or rituximab [11]. 

The prognosis is generally favorable in patients with IgA vasculitis presenting with arthralgia and mild renal involvement [3,5,8]. Arthralgia, often transient and non-deforming, typically resolves without long-term sequelae. Similarly, mild renal involvement characterized by microscopic hematuria and proteinuria at subnephrotic levels tends to have a good prognosis, with most cases resolving spontaneously without progressing to severe renal impairment [8]. However, regular monitoring is essential to detect any potential worsening of renal function or relapses. Overall, the prognosis in these patients is favorable, with the majority experiencing resolution of symptoms and maintaining good long-term renal function [19-21].

## Conclusions

The presented case underscores the importance of a thorough clinical assessment, incorporating detailed history-taking, physical examination, and judicious use of diagnostic tests to navigate the diagnostic uncertainties associated with vasculitis. Despite inconclusive histological findings from skin and renal biopsies, the clinical presentation, including palpable purpura, constitutional symptoms, and renal involvement, strongly supported the diagnosis of IgA vasculitis. The delayed biopsies may have contributed to the lack of definitive histological features, highlighting the importance of timely sampling during active disease phases.

Multidisciplinary collaboration, as demonstrated in this case, is essential for comprehensive patient care, integrating expertise from specialties such as nephrology, dermatology, and internal medicine. Despite diagnostic and therapeutic uncertainties, a meticulous approach guided by clinical judgment and supported by evidence-based practices remains paramount in managing IgA vasculitis effectively and improving patient outcomes.
